# Rapid Detection of Echinocandins Resistance by MALDI-TOF MS in *Candida parapsilosis* Complex

**DOI:** 10.3390/microorganisms8010109

**Published:** 2020-01-13

**Authors:** Ana Emília M. Roberto, Danilo E. Xavier, Esteban E. Vidal, Cláudia Fernanda de L. Vidal, Rejane P. Neves, Reginaldo G. de Lima-Neto

**Affiliations:** 1 Graduate Program in Fungal Biology, Federal University of Pernambuco (UFPE), Recife-PE 50.740-600, Brazil; ranamedeiros@hotmail.com; 2 Instituto Aggeu Magalhães, FIOCRUZ, Recife-PE 50.670-420, Brazil; danilo.xavier@cpqam.fiocruz.br; 3 Center for Strategic Technologies Northeastern (CETENE), Recife-PE 50.740-545, Brazil; esteban.espinosa.vidal@gmail.com; 4 Hospital of Clinics, Recife-PE 50.670-901, Brazil; vidal.claudia@gmail.com

**Keywords:** mass spectrometry, susceptibility testing, candidemia, resistance

## Abstract

Mass spectrometry by matrix-assisted laser desorption/ionization time-of-flight (MALDI-TOF) was used to identify and differentiate the pattern of susceptibility of clinical isolates of *Candida parapsilosis* complex. 17 *C. parapsilosis sensu stricto*, 2 *C. orthopsilosis,* and 1 *C. metapsilosis* strains were obtained from blood cultures, and three different inocula (10^3^, 10^5^, and 10^7^ CFU/mL) were evaluated against three echinocandins at concentrations ranging from 0.03 to 16 µg/mL after incubation of 1 h, 2 h, and 3 h. Drug-free control was used. The spectra obtained at these concentrations were applied to generate composite correlation index (CCI) matrices for each yeast individually. After cross correlations and autocorrelations of each spectra with null (zero) and maximal (16) concentrations, the CCI was used as separation parameter among spectra. Incubation time and inoculum were critical factors to reach higher precision and reliability of this trial. With an incubation time of 3 h and inoculum of 10^7^ CFU/mL, it was possible to determine the breakpoint of the clinical yeasts by MALDI-TOF that presented high agreement with the clinical laboratory standard institute (CLSI) reference method. Herein, we show that mass spectrometry using the MALDI-TOF technique is powerful when it exploits antifungal susceptibility testing assays.

## 1. Introduction

Incidence of invasive fungal infections (IFIs) has tragically increased during the last twenty years, despite advances in methods of diagnosis [1,2]. *Candida*-related IFIs are linked to elevated morbidity and mortality, as well as a high cost of healthcare [1,2]. *Candida albicans* is still the major species related to candidemia wide world; however, the diversity of other *Candida* species differs according geographical location. Thus, Brazilian distribution displays a high frequency of *C. parapsilosis* as the typical etiologic species [3,4,5].

Invasive candidiasis presents unspecific clinical symptoms, which make early diagnosis difficult, requiring blood cultures that are time-consuming and have little sensitivity [6]. Therefore, in a substantial number of cases, the diagnosis of candidemia is usually established late or even at autopsy [7]. In this sense, the general consensus assigns earlier initiation with precise antifungal treatments, proportionally increasing the patient survival. Thus, it is crucial to develop new rapid diagnostic methods capable of efficiently detecting IFIs at the beginning of the course of the disease, with high sensitivity and specificity [8]. At the same time, the susceptibility profiles against antifungal drugs vary greatly between species, being equally necessary to the implementation of a rapid antifungal susceptibility test. 

Few developments in microbiological diagnostics have had a rapid impact on the identification of microorganism species as the matrix-assisted laser desorption/ionization time-of-flight mass spectrometry (MALDI-TOF MS) technique. Spectra generated are analyzed as a fingerprint where ribosomal proteins appear with molecular mass ranging from 2000–20,000 Da, which is an advantage due to the fact they may be easily employed as biomarkers. After spectra acquisition, MALDI-TOF MS-based microorganism identifications are reached by comparing their protein fingerprints with super spectra and reference spectra included in database of well-characterized isolates from renowned culture collections from around the world [9,10].

This approach has been applied to the detection of *Candida* yeasts that have acquired resistance to antifungal therapy [9,11,12]. Proteomics changes that occur in yeasts after exposition to antifungal drugs enable the determination of the minimal profile change concentration (MPCC), a breakpoint that may become a more accurate preference method rather than usual minimum inhibitory concentration (MIC) obtained by clinical laboratory standard institute (CLSI) [13,14,15]. 

Considering the aforementioned, the objective of this study was to development an ultra-fast and high accuracy echinocandins-susceptibility diagnosis method for *Candida parapsilosis* complex clinical strains based on the MALDI-TOF MS system; comparing the results with the current CLSI protocol.

## 2. Materials and Methods

### 2.1. Specimen, Diagnosis and Storage

Blood samples were collected after Human Ethics Committee approval of the Federal University of Pernambuco (UFPE), Brazil, under identification number CAAE 58601316.5.0000.5208. Approval was granted on 13 June 2016, under number 1,586,343. Venous blood samples were aseptically collected three times on consecutive days and incubated at 37 °C in bottles with brain heart infusion media for isolation of the clinical strains. Fifty-nine *Candida*-positive blood samples were obtained from inpatients of the intensive care unit (ICU) of public hospital at the Northeast from the Brazil among June 2016 to September 2018.

Spectra of clinical strains were obtained according to Lima-Neto et al. [10] with modifications. The MALDI TOF Autoflex III Mass Spectrometer (Bruker Daltonik GmbH, Bremen, Germany) set up with a 1064 nm laser of neodymium crystal (Nd:Y_3_Al_5_O_12_) to 66% power was used. The linear mode with a 104 ns pulsed laser and an acceleration voltage of +20 kV was used to register the mass range between 2000 to 20,000 Da. The peak lists obtained were exported to the software package MALDI Biotyper™ 3.0 (Bruker Daltonik GmbH, Bremen, Germany), where identifications were achieved. 

A representative of each species of the complex was stocked in URM Culture Collection at the UFPE. This collection is registered in the WDCM of the WFCC as “604” under the acronym URM (University Recife Mycology).

### 2.2. Antifungal Susceptibility Testing (AFST) by CLSI Method

Reference microdilution trials M27-A3 and M27-S4 guidelines [13,14] were used. The standard RPMI 1640 medium (Sigma-Aldrich, St. Louis, MO, USA) was buffered to pH 7.0 ± 0.1 with 0.165 M of morpholino propane sulfonic acid (MOPS; Sigma-Aldrich, St. Louis, MO, USA). RPMI 1640 medium sterilization by filtration with membrane 0.22 (Millipore, Darmstadt, Germany) was performed. Antifungal drugs were dissolved and stored in dimethyl sulfoxide (DMSO, Synth, Diadema, São Paulo, Brazil) at 1600 μg/mL. For testing, caspofungin (MSD, Riom, France), anidulafungin (Pfizer, NY, EUA), and micafungin (Astellas Pharma Inc, Tokyo, Japan) were diluted and used at concentrations among 0.03 to 16 μg/mL. 

*Candida parapsilosis* complex isolates were subcultured on sabouraud dextrose agar (SDA) with yeast extract, and incubated at 35 °C for 24-h. One to three single colonies were suspended in 0.85 g/L NaCl, and inoculum containing 1.0 to 5.0 × 10^6^ CFU/mL was adjusted in a spectrophotometer at 530nm to obtain 90% transmittance. Each inoculum was two-fold diluted to reach 10^3^ CFU/mL.

Microtiter plates of flat 96-well (TPP, Trasadingen, Switzerland) were used. After inoculum was added to the wells containing the serial dilutions of antifungal agents, the plates were incubated at 35 °C for 24 h to define the minimum inhibitory concentration (MIC). The test was carried out in duplicate. The antifungal testing according to CLSI classifies the strains of *C. parapsilosis* complex against anidulafungin, caspofungin, and micafungin as susceptible with MIC ≤2 µg/mL, intermediate at 4 µg/mL, and resistant at ≥8 µg/mL [14].

*Candida parapsilosis* ATCC 22019 was used as quality control as recommended by CLSI.

### 2.3. Antifungal Susceptibility Testing by MALDI-TOF MS (AFST-MS)

The AFST-MS was carried out in accordance with Marinach et al. [11] and modifications by De Carolis et al. [16]. Protein extraction of the yeasts was performed from three different concentrations of inoculum suspensions of 10^3^, 10^5^, and 10^7^ CFU/mL, cultivated in RPMI containing serial dilutions (0.03125 to 16 μg/mL) of the three echinocandins, separately in microtiter plates under stirring for 1 h, 2 h, and 3 h of incubation maintained at 37 °C. Each suspension was centrifuged twice to 12,000× *g* for 2 min with posterior washing in deionized water. After third washing, the cell pellet was resuspended in 10% formic acid and centrifuged. One µL of this supernatant was directly spotted in duplicate on target plate, and then 1 µl of absolute ethanol was added along with 1 µL of a saturated solution acid α-cyano-4-hydroxycinnamic acid in 50% acetonitrile–2.5% trifluoroacetic acid, allowing for complete drying.

Protein spectra were acquired with the same MALDI equipment described above; however, mass range used was 2000–10,000 Da. For each assay, spectrum was analyzed with the software FlexControl™ (Bruker Daltonick GmbH, Bremen, Germany).

### 2.4. Data Analysis

Statistical software composite correlation index (CCI) from the Biotyper™ (Bruker Daltonick GmbH, Bremen, Germany) was used to examine the relationship among each spectra according to reported by Marinach et al. [11] and modified by De Carolis et al. [16].

Compositions of cross correlations and autocorrelations of the yeasts were used as a separation parameter among the spectra. CCI values near 1 represent a high similarity conformance of spectra profile, while CCI values around 0 indicate low similarity. The results are displayed in a correlation matrix and posteriorly in a heat map. Proteins spectra closely related are marked in hot colors, while unrelated ones are in cold colors. This enables one to define the MPCC for anidulafungin, caspofungin, and micafungin, the value defined as the lowest drug concentration where a change detectable in the MALDI-TOF MS profile occurs. 

## 3. Results

Among the 59 patients diagnosed with candidiasis, twenty were diagnosed as *Candida parapsilosis* complex by MALDI-TOF MS, and were selected for AFST by both microdilution method and mass spectrometry methodologies. Seventeen yeasts were *Candida parapsilosis sensu stricto*, two were *Candida orthopsilosis,* and one was *Candida metapsilosis*. The other yeasts isolates were 16 *Candida albicans*, 13 were *Candida glabrata,* and 10 were *Candida tropicalis.*

The CLSI-based AFST method showed that all 20 clinical isolates of *Candida parapsilosis* complex were susceptible against caspofungin and micafungin and one *Candida parapsilosis sensu stricto* was resistant against anidulafungin only. According to AFST-MS, all 20 clinical isolates were susceptible to anidulafungin and micafungin and one *Candida orthopsilosis* was intermediate against caspofungin with inoculum suspension of 10^7^ CFU/mL after incubation time of 3 h. Inoculum suspensions of 10^3^ and 10^5^ CFU/mL, besides having an incubation time of 1 h or 2 h, did not produce detectable peaks on spectra. The breakpoints and their MIC or MPCC values obtained by antifungal susceptibility testing carried out by CLSI or MALDI-TOF, respectively, are summarized in Table 1. The two main discrepancies are highlighted in green letters.

Using the CLSI-based AFST microdilution method as reference on antifungal evaluation, the breakpoints obtained with AFST-MS showed concordance of 95%, 95%, and 100% for anidulafungin, caspofungin, and micafungin, respectively, among all isolates analyzed. 

**Figure 1:**
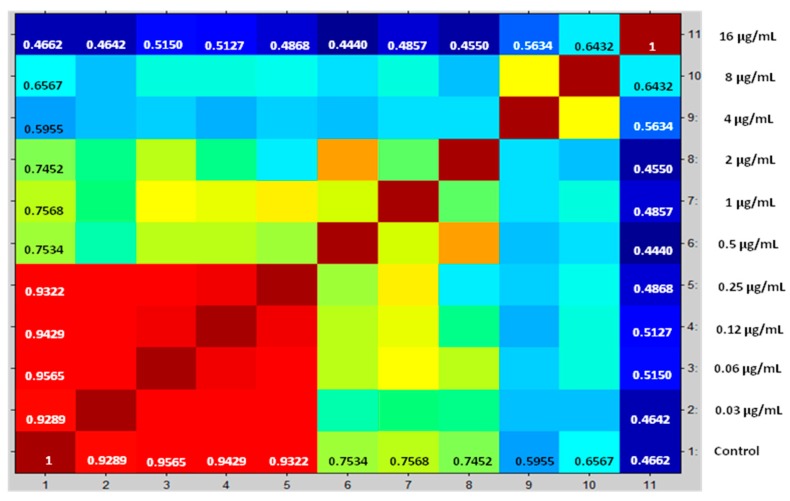
Heat map translated from mass spectra selected of *Candida parapsilosis* 31 exposed against caspofungin for 3h and used to generate CCI matrix. Red gradients coincide with strong associations and green to deep-blue gradients coincide with weak associations. Spectra obtained below MPCC showed higher similarity with the null drug treatment. Spectra obtained at and above MPCC are very similar to extreme drug concentration. *X*-axis numbers have the same drug concentration on the *y*-axis. MPCC = 0.5 µg/mL. Source Maldi Biotyper 3.0.

**Figure 2:**
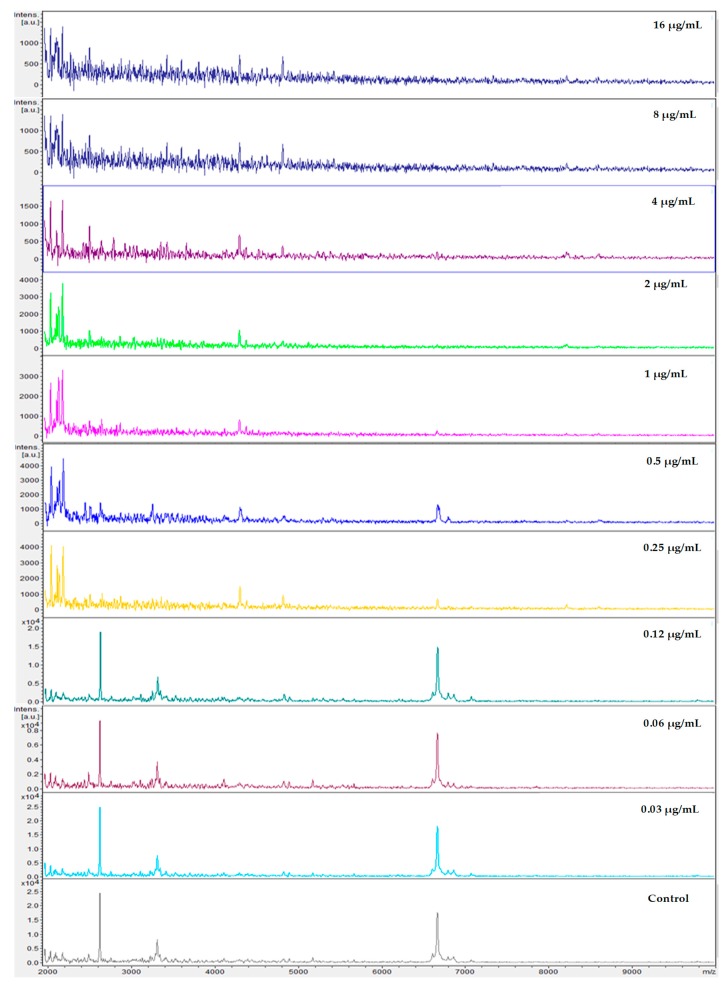
MALDI-TOF spectra profiles obtained from *Candida parapsilosis* 31 under ten different concentrations of caspofungin and control (no drug) after exposure of 3 h at 37 °C. Minimal spectra alterations are visible following exposure to 0.5 μg/mL. Source: MALDI FlexAnalysis™ (Bruker Daltonics, Bremem, Germany).

**Figure 3:**
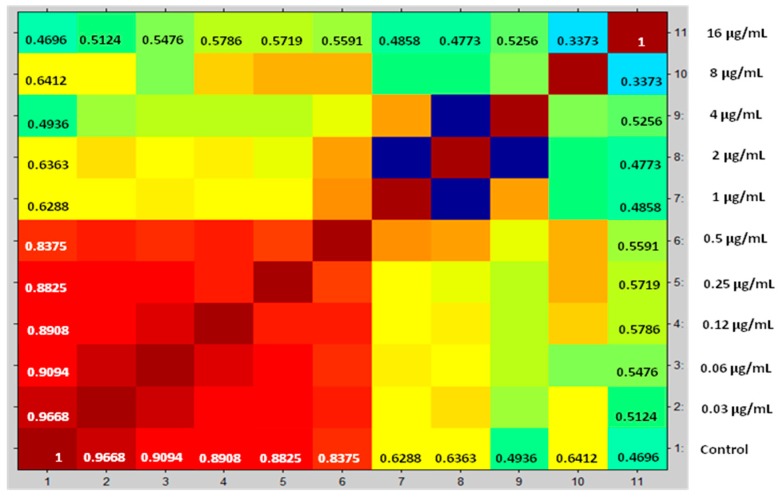
Heat map translated from mass spectra selected of *Candida parapsilosis* 31 exposed against anidulafungin for 3h and used to generate CCI Matrix. Red gradients coincide with strong associations and yellow to green gradients coincide with weak associations. Spectra obtained below MPCC showed higher similarity with the null drug treatment. Spectra obtained at and above MPCC shower significant similarity with the extreme drug concentration. *X*-axis numbers have the same drug concentration on the *y*-axis. MPCC = 1 µg/mL. Source Maldi Biotyper 3.0.

**Figure 4:**
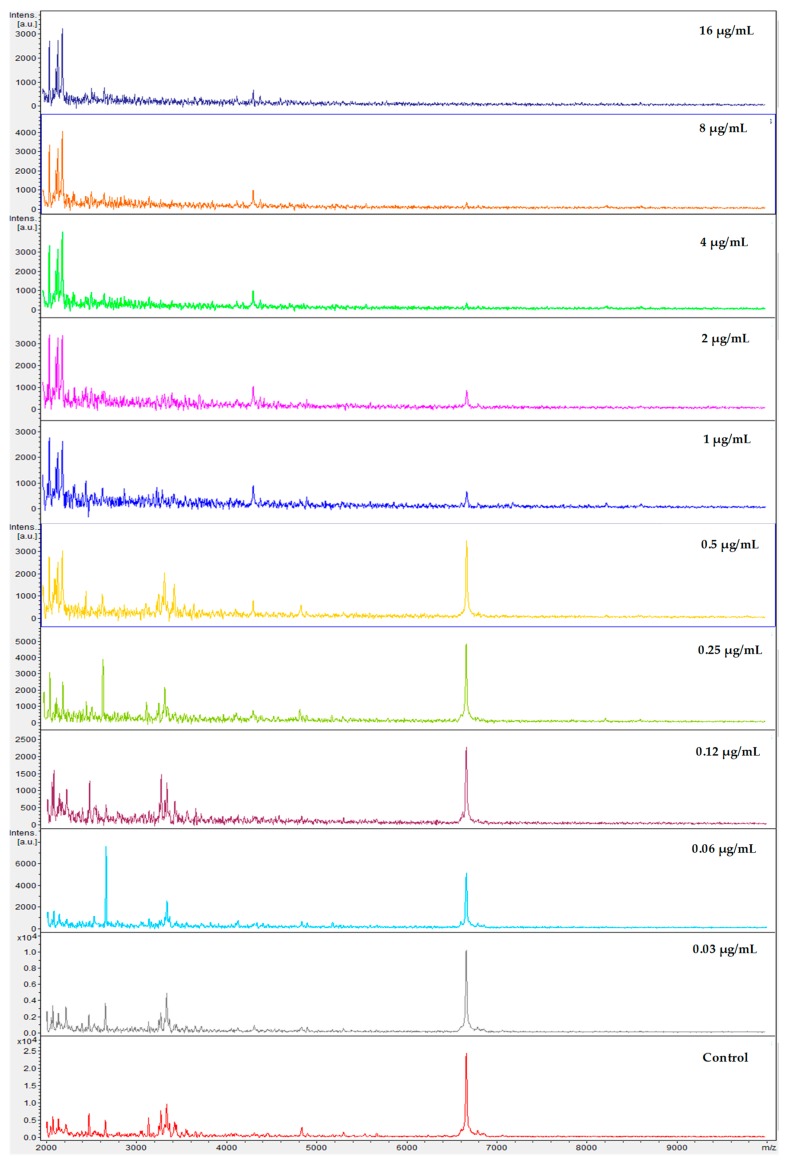
MALDI-TOF spectra profiles obtained from *Candida parapsilosis* 31 under ten different concentrations of anidulafungin and control (no drug) after exposure of 3 h at 37 °C. Minimal spectra alterations are visible following exposure to 1 μg/mL. Source: MALDI FlexAnalysis™ (Bruker Daltonics, Bremem, Germany).

**Figure 5:**
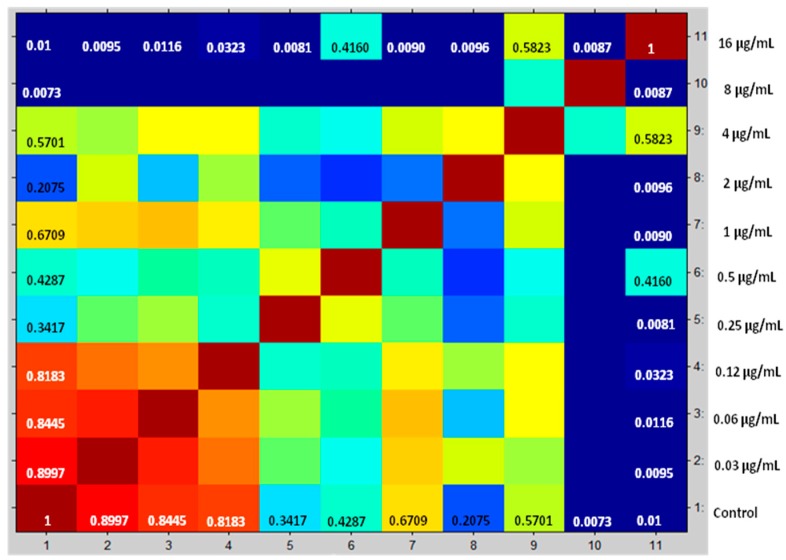
Heat map translated from mass spectra selected of *Candida parapsilosis* 31 exposed against micafungin for 3h and used to generate CCI Matrix. Red gradients coincide with strong associations and green to deep-blue gradients coincide with weak associations. Spectra obtained below MPCC were more similar to the null drug treatment. Spectra obtained at and above MPCC were very similar to the extreme drug concentration. *X*-axis numbers have the same drug concentration on the *y*-axis MPCC = 0.25 µg/mL. Source Maldi Biotyper 3.0.

**Figure 6:**
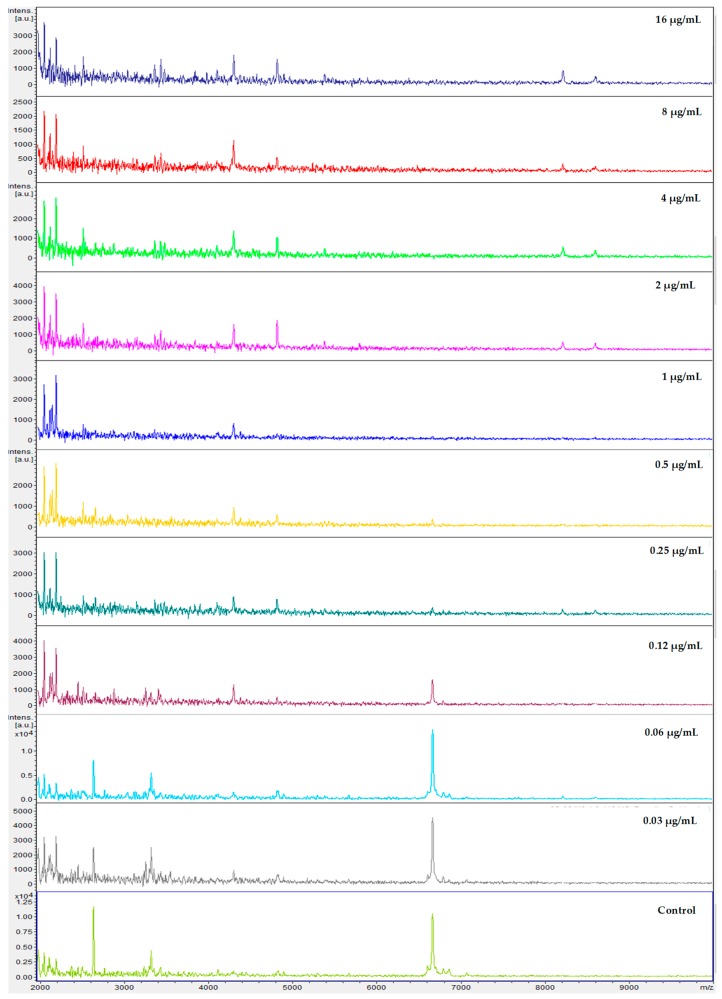
MALDI-TOF spectra profiles obtained from *Candida parapsilosis* 31 under ten different concentrations of micafungin and control (no drug) after exposure of 3 h at 37 °C. Minimal spectra alterations are visible following exposure to 0.25 µg/mL. Source: MALDI FlexAnalysis™ (Bruker Daltonics, Bremem, Germany).

After correlation of every spectrum obtained after exposure to the concentrations 0.03–8 µg/mL with one of the two extreme concentrations, 16 (maximum) or 0 (null), the derived CCI matrixes and their respective heat map for all yeasts of the *Candida parapsilosis* complex were generated. These results were expressed in tables for each echinocandin tested (data not entirely shown). For example, we displayed the results for *Candida parapsilosis sensu stricto* 31, which was classified for both methods as susceptible. The CCI matrix, their heat map, and spectra subset are presented, respectively, in Table 2, Figure 1 and Figure 2 for caspofungin; Table 3, Figure 3 and Figure 4 for anidulafungin; and Table 4, Figure 5 and Figure 6 for micafungin.

## 4. Discussion

The mass spectrometry by MALDI-TOF enables one to visualize the variations among the ionized protein composition from strains with different breakpoints by the comparison of their spectra. Our results are in accordance with Vella et al. [12], Saracli et al. [9], Vella et al. [15], and Paul et al. [17], which affirm that after exposition of yeasts against different concentrations of the antifungal drugs it is possible to find changes in the peak distribuition in spectra of every *Candida* tested. Herein, we exploit this potential of MALDI-TOF MS with the second most occurent agent of candidemia in the Latin America: *Candida parapsilosis* complex [1,4]. To the best of our knowledge, this is first work that analyzes antifungal susceptibility profile of *Candida parapsilosis* complex species by MALDI-TOF. Furthermore, echinocandins is extensively used in critically ill patients with suspected or confirmed candidemia by *Candida parapsilosis*. We must point out that Vella et al. [12] and De Carolis et al. [16] brought us new knowledge in this field using caspofungin, and Vella et al. [16] using anidulafungin. However, there have been no publications with micafungin so far.

The traditional antifungal susceptibility testing by CLSI may detect resistance of a microorganism independently of its mechanism. Therefore, this detection by mass spectrometry may occur if the amount of ionized protein is expressed sufficiently by the yeast cells evaluated [18]. Comparing the CLSI-based AFST and the AFST MS methodologies, it was possible to find the high agreement level, reaching a performance of 100% for micafungin. It is important to highlight that the yeasts evaluated in this research were obtained from patients with candidemia admitted in intensive care unit and selected irrespective of their susceptibility or kind of drug resistance mechanisms.

Paul et al. [17] used recently CCI-based proteomic study to analyze 15 *C. tropicalis* fluconazole-resistant and 19 *C. tropicalis* fluconazole-susceptible in comparison with international method published by CLSI microdilution assay. According to researches, the correlation between MPCC and MIC was significant and AFST-MS assay may be used as a rapid screening technique for fluconazole resistance in *C. tropicalis.*

Vella et al. [15] evaluated this physical-chemical technique using a subset of 80 *C. glabrata* clinical strains after 3h incubation with anidulafungin. When comparing changes on spectra to the standard method recommended by the CLSI, 57/58 (100%) isolates with WT sequence were classified as susceptible, but 11/22 (50.0%) resistant isolates were really classified as resistant to anidulafungin. The aforementioned authors have repeated the assay for the 11 resistant isolates with longer incubation times (6, 9, and 12 h); however there were two unresolved mistakes for anidulafungin. After 15 h incubation, 100% essential agreement was achieved [15].

Currently, the AFST-MS provides MPCC breakpoints like EUCAST and CLSI provides MIC. This new MALDI-TOF-based antifungal susceptibility testing was shortened by several authors reported here as Vella et al. [12], Saracli et al. [9], and Vella et al. [15]. However, we opted to evaluate MPCC with extended drug concentrations (0.03–16 µg/mL) to determine maximal discrepancy in relation to dilutions, and we verified that our results were similar to those reported byEspinel-Ingroff et al. [18], who advocate a cutoff value of two dilutions between MPCC and MIC, for maximal acceptance of disagreement. Due to extended drug concentration evaluated, we have not tested a large number of strains at this time.

Our group also performed tests with a decreased incubation time (1 and 2 h), but we did not get positive results. Nowadays, new tests have been carried out involving echinocandins with changes in the methodology, like inocula concentration and cultured media, in order to make tests more reproducible and faster. This is relevant, since antifungal susceptibility profile is shown to be more resistant in this species against this antifungal class.

All in all, we demonstrate that the readings by mass spectrometry using MALDI-TOF represent a timesaving (3 h versus 24 h) in comparison with CLSI microdiluition trial. In addition, antifungal susceptibility testing by CLSI and MALDI-TOF methods showed concordance of 95% for anidulafungin and caspofungin, besides 100% for micafungin. This proteomic technique presents advantage of excluding subjective read-outs, which occur with the CLSI method when fungi are evaluated. Pharmacoeconomics should also be carefully analyzed to confirm that MALDI-TOF is more cost-effective [17,19].

## 5. Conclusions

Herein, the technology MALDI-TOF MS displayedwas shown to be effective when applied to the AFST trials. Incubation time and inocula concentration were crucial to reach a maximum level of agreement with a standard test. We reinforced the fact that MALDI-TOF is a faster alternative to traditional methods. However, AFST-MS needs to be further investigated by researchers and further surveyed to be optimized, and reproduced in a laboratory setting.

## Figures and Tables

**Table 1:** Comparison between AFST * from CLSI ** and AFST from MALDI-TOF MS ^¶^ performed after 3h exposure of 20 clinical isolates of the *Candida parapsilosis* complex against three echinocandins.

Isolated and Lab Number	Breakpoint (MIC in µg/mL) Breakpoint (MPCC in µg/mL)					
	Anidula.	Caspo.	Mica.	Anidula.	Caspo.	Mica.
*C. parapsilosis* 346A	S (0.25)	S (0.25)	S (2)	S (0.5)	S (0.125)	S (0.5)
*C. parapsilosis* 346B	S (0.25)	S (0.25)	S (2)	S (0.25)	S (0.0625)	S (0.5)
*C. parapsilosis* 474	S (0.5)	S (0.5)	S (2)	S (0.125)	S (0.5)	S (0.5)
*C. parapsilosis* 595	S (0.25)	S (0.25)	S (1)	S (0.0625)	S (0.25)	S (0.25)
*C. parapsilosis* 596A	S (0.25)	S (0.25)	S (0.5)	S (0.0625)	S (0.125)	S (0.5)
*C. parapsilosis* 596B	S (0.25)	S (0.25)	S (0.5)	S (0.125)	S (0.0625)	S (2)
*C. parapsilosis* 5902	S (0.0625)	S (0.0625)	S (0.03125)	S (0.25)	S (0.25)	S (0.03125)
*C. parapsilosis* 12	S (0.125)	S (0.03125)	S (0.03125)	S (0.03125)	S (0.125)	S (0.06125)
*C. parapsilosis* 29	S (0.125)	S (0.0625)	S (0.0625)	S (0.0625)	S (0.25)	S (0.25)
*C. parapsilosis* 30	S (0.0625)	S (0.0625)	S (0.0625)	S (0.125)	S (0.25)	S (0.25)
*C. parapsilosis* 31	S (0.25)	S (0.125)	S (0.0625)	S (1)	S (0.5)	S (0.25)
*C. parapsilosis* 39	S (0.0625)	S (0.125)	S (0.0625)	S (0.03125)	S (0.5)	S (0.25)
*C. parapsilosis* 40	**R (16)**	S (0.25)	S (2)	**S (1)**	S (0.5)	S (2)
*C. parapsilosis* 44	S (0.03125)	S (0.0625)	S (0.0625)	S (0.03125)	S (0.25)	S (0.25)
*C. parapsilosis* 45	S (0.25)	S (0.0625)	S (0.0625)	S (0.125)	S (0.0625)	S (0.25)
*C. parapsilosis* 48	S (0.0625)	S (0.0625)	S (0.03125)	S (0.25)	S (0.25)	S (0.03125)
*C. parapsilosis* 49	S (0.03125)	S (0.03125)	S (0.06125)	S (0.03125)	S (0.125)	S (0.25)
*C. orthopsilosis* 03	S (0.0625)	**S (0.125)**	S (0.5)	S (0.25)	**I** ^+^ **(4)**	S (0.5)
*C. orthopsilosis* 07	S (2)	S (0.0625)	S (0.5)	S (0.03125)	S (0.03125)	S (0.03125)
*C. metapsilosis* 32	S (1)	S (1)	S (0,5)	S (0.25)	S (0.25)	S (0.5)
ATCC 22019	S (0.5)	S (0.5)	S (1)	S (0.5)	S (0.25)	S (0.5)

* AFST—antifungal susceptibility testing. ** CLSI—clinical laboratory standard institute. ^¶^ MALDI-TOF MS—matrix-assisted laser desorption/ionization time-of-flight mass spectrometry. MIC—minimal inhibitory concentration obtained by CLSI method. MPCC—minimal profile change concentration obtained by MALDI-TOF MS technique. S—susceptible. R—resistant. ^+^ I—intermediate.

**Table 2:** Composite correlation index (CCI) matrix created from the correlations of all spectral intervals values of the *Candida parapsilosis* 31 exposed to caspofungin. MPCC was quantitatively determined by statistical relation between whole data set obtained by acquired spectra.

MPCC (µg/mL)	CCI Null	CCI Maximum
0.0000	0.4662	1.0000
0.0313	0.4642	0.9289
0.0625	0.5150	0.9565
0.1250	0.5127	0.9429
0.2500	0.4868	0.9322
0.5000	0.4440	0.7534
1.0000	0.4857	0.7568
2.0000	0.4550	0.7452
4.0000	0.5634	0.5955
8.0000	0.6432	0.6567
16.0000	1.0000	0.4662

**Table 3:** CCI matrix created from the correlations of all spectral intervals values of the *Candida parapsilosis* 31 exposed to anidulafungin. MPCC was quantitatively determined by statistical relation between whole data set obtained by acquired spectra.

MPCC (µg/mL)	CCI Null	CCI Maximum
0.0000	0.4696	1.0000
0.0313	0.5124	0.9668
0.0625	0.5476	0.9094
0.1250	0.5786	0.8908
0.2500	0.5719	0.8825
0.5000	0.5591	0.8375
1.0000	0.4858	0.6288
2.0000	0.4773	0.6363
4.0000	0.5256	0.4936
8.0000	0.3373	0.6412
16.0000	1.0000	0.4696

**Table 4:** CCI matrix created from the correlations of all spectral intervals values of the *Candida parapsilosis* 31 exposed to micafungin. MPCC was quantitatively determined by statistical relation between whole data set obtained by acquired spectra.

MPCC (µg/mL)	CCI Null	CCI Maximum
0.0000	0.0100	1.0000
0.0313	0.0095	0.8997
0.0625	0.0116	0.8445
0.1250	0.0323	0.8183
0.2500	0.0081	0.3417
0.5000	0.4160	0.4287
1.0000	0.0090	0.6709
2.0000	0.0096	0.2075
4.0000	0.5823	0.5701
8.0000	0.0087	0.0073
16.0000	1.0000	0.0100
